# When Are We Most Vulnerable to Temperature Variations in a Day?

**DOI:** 10.1371/journal.pone.0113195

**Published:** 2014-12-02

**Authors:** Chao-Yu Guo, Wen-Chi Pan, Mu-Jean Chen, Chen-Wei Tsai, Nai-Tzu Chen, Huey-Jen Su

**Affiliations:** 1 Division of Biostatistics, Institute of Public Health, National Yang Ming University, Taipei, Taiwan; 2 Aging and Health Research Center, National Yang Ming University, Taipei, Taiwan; 3 Biostatistical Consulting Center, National Yang Ming University, Taipei, Taiwan; 4 Department of Environmental and Occupational Health, College of Medicine, National Cheng Kung University, Tainan, Taiwan; 5 Department of Epidemiology, School of Public Health, Brown University, Providence, Rhode Island, United States of America; 6 National Environmental Health Research Center, National Health Research Institutes, Miaoli, Taiwan; National Taiwan University, Taiwan

## Abstract

Daily temperature measures are commonly used when examining the association between temperature and mortality. In fact, temperature measures are available 24 hours a day and more detailed records may provide a better prediction of mortality compared to daily statistics. In this article, monthly stratified analysis modeling for mortality is conducted for the total population as well as the stratified elderly and younger subgroups. We identified the most significant time during the day that is associated with daily mortality. Surprisingly, the estimates of relative risk and magnitude of associations derived from the hourly temperature measures are similar or even stronger compared to those modeled by the daily statistics. This phenomenon remains true for lagged hourly temperature measures and the changing patterns of associations from January through December are revealed. In summary, people are the most vulnerable to temperature variations in the early morning around 5 am and the night time around 8 pm.

## Introduction

Studies in several countries have suggested that either hot or cold temperatures may significantly increase daily mortality rates [1]–[14]. In addition, people who live in colder places are less affected by cold weather [1], [7], while those in hotter climates are better adapted to extreme heat [12], [15]. High winter mortality during cold temperatures was also reported in a subtropical city, Guangzhou, China [16]. Recently, susceptibility to mortality during extreme weather has also been discussed [17].

A distinctive pattern of temperature is that it recurs daily, and the range of temperature during the day, which is measured by the difference between the daily maximum and minimum temperature, can be quite broad. The daily maximum temperature usually occurs in the middle of the day, which often coincides with the peak time for outdoor activity. In contrast, the daily minimum temperature is usually measured at night when most people are indoors. Generally speaking, the daily mean temperature, which is an average of multiple observations in the same day, is thought to be a good estimate of exposure and less affected by measurement errors compared with other temperature data, has been shown to be associated with mortality [1], [3], [4], while others have examined impact of the daily minimum and maximum temperature [9].

The popular distributed lag model [18]–[19] examines time series data in which a regression equation is used to predict current values of a dependent variable based on both the current values of an explanatory variable and the lagged (past period) values of the explanatory variable. The application of the distributed lag non-linear model [20] was used to identify mortality risks based on all causes, including circulatory and respiratory diseases for the elderly in Taiwan [21]. Adjusting for the monthly effect, the relationship between the heat index and mortality in 6 major cities in Taiwan was identified [22].

Although the temperature data are measured hourly, the mortality data are still recorded daily in our database. If the hourly mortality is available, the distributed lag model could be implemented using 24-hourly temperature measures as the joint predictors. However, mortality is recorded daily and the distributed lag model may not be the optimal method for such data structure. Nevertheless, even if the hourly mortality is available and the distributed lag model is fitted to the hourly temperature measures, the interpretation is the overall temperature effect in the past 24 hours to the current hourly mortality. Since the aim of this study is to discover the specific time when people are most vulnerable to temperature variations during their daily life, we implement Poisson regression using generalized linear model for each hourly temperature measure.

## Materials and Methods

### Study area

**Figure 1:**
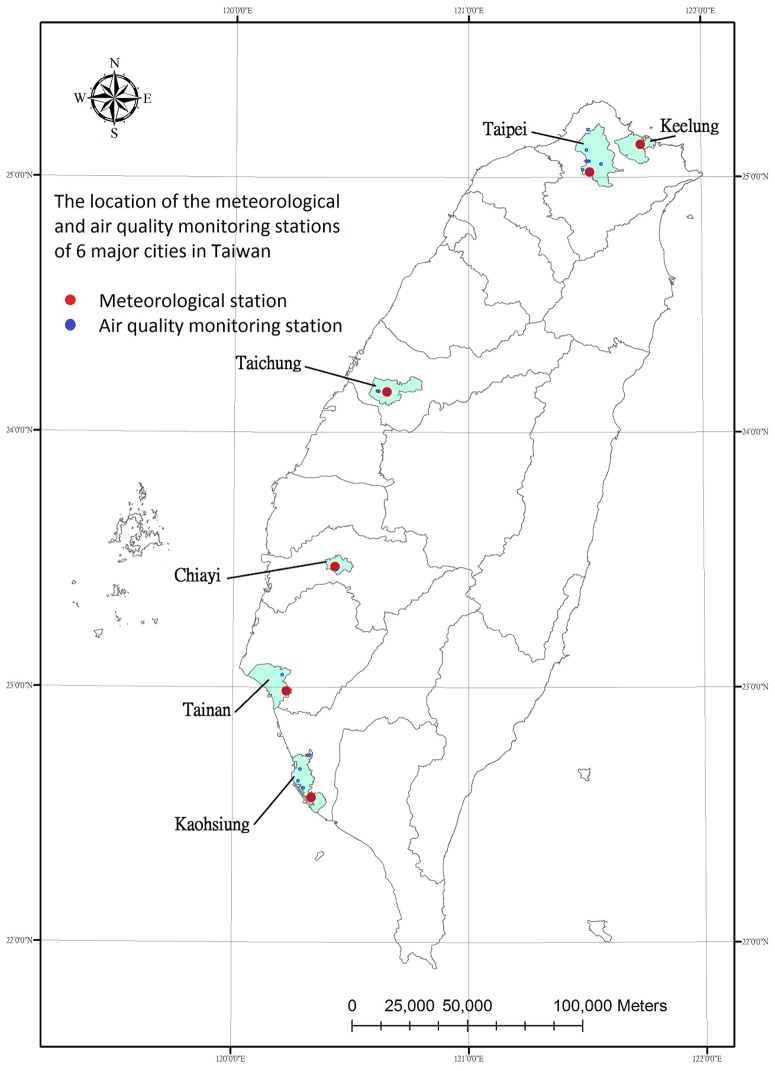
The locations of meteorological and air pollution monitoring stations in 6 major cities in Taiwan.

This study carries out monthly stratified analysis and demonstrates various impacts of temperature measures on mortality among different groups of residents of all ages, as well as the younger group (population aged 64 years or younger) and elderly (population aged 65 years or older) people in 6 major cities (Keelung, Taipei, Taichung, Chiayi, Tainan, and Kaohsiung) in Taiwan from 1994 to 2008. The locations of the 6 major cities studied in Taiwan are shown Figure 1.

### Mortality data

In Taiwan, all deaths are reported to the township and district household registry office; the National Death Registry database was obtained from the Department of Health (with no personal information involved). Vital statistics contained underlying cause-of-death, age, sex, place of death and household registration. The total non-accidental causes mortality rate (per 100,000) for 6 cities was estimated using the number of deaths due to non-accidental illness (ICD-9: 001–799; ICD-10: A00-R99) as the numerator and the total population in the corresponding region as the denominator. Mortality included the death rate of the total population, the younger population and elderly population. Because the National Death Registry database is a secondary database without detailed personal information (e.g. ID number and address), all data were analyzed anonymously.

### Meteorological and air quality data

The 24 hour minimum, mean and maximum ambient temperature and relative humidity data of the monitoring stations of the Taiwan Central Weather Bureau (CBW) from six cities were acquired from 1994 to 2008. The hourly concentrations of air polluters (O_3_, PM_10_ and PM_2.5_) of the monitoring stations of the Environmental Protection Agency (EPA) of Taiwan from six cities were also acquired from 1994 to 2008. The ArcGIS 9.3 system was used to denote the study areas and map the locations of monitoring stations. The distribution of meteorological and air pollution monitoring stations of 6 major cities in Taiwan are shown in Figure 1.

### Statistical analysis

Since the distributed lag model that utilize 24 hourly measures in one model is not the optimal method for the hourly temperature data, the main association between hourly temperature measures and daily mortality was assessed by Poisson regression models. Let Y be the daily mortality, we have 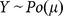. As a result, the main model is 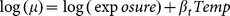, where “Temp” is the hourly temperature measure and “log(exposure)” is the offset. Since 24 models were fitted separately for each hourly temperature measure, the issue of co-linearity using 24 hourly statistics is avoided.

Daily temperature measures (mean, minimum, maximum, and range) as well as the hourly mean temperatures were the primary predictors analyzed as continuous variables. Models were adjusted for air pollutants, city effects, calendar year, daily relative humidity, and holidays. For air pollutant data, including all daily statistics such as minimum, maximum, mean, and range, the maximum PM_10_ and mean O_3_ were adjusted in the model, since they have the most significant association with daily mortality in our data. Different daily PM_10_ and O_3_ were analyzed as the sensitivity analysis. However, the effect estimates are quite similar. Take the O_3_ for an example, the most significant association is in June for both daily maximum and mean O_3_. The corresponding results are displayed in Table S1. Regarding the city characteristics, researchers could perform time-series analysis for one city with temporal correlations and then apply meta-analysis to combine the effects of several cities. However, Taiwan is relatively small and stratified analyses of the six cities yielded very similar results. Therefore, instead of the meta-analysis approach, the city effect was considered as a fixed effect in the Poisson model. Relative risk could be estimated since we employed a natural logarithm to transform the population size of each city. Because the lag effects of hot, cold, the daily maximum, minimum, and mean temperature on morbidity have been extensively studied to date, these statistics were included in the analysis. Sixty-five years-old was the cutoff point to define the subgroups and the differentiated effects of temperature on daily mortality among the younger and elder populations were modeled separately.

Conventionally, the seasonal effect is either adjusted in the statistical model or the analysis is stratified by the four seasons. However, we discovered extremely significant interactions between the seasonal effect and daily temperature measures when correlating daily mortality in Taiwan. The interactions between the seasons and daily temperature measures on mortality were assessed by the interaction P-values, which were calculated based on comparisons between the full model (with interaction terms of season by temperature) and the reduced model (with season and temperature as the main effect factors).

In Table 1, all daily temperature statistics are indicated in the first column, adjustments for PM_10_ and O_3_ are indicated in the second column, and stratifications by the four seasons are shown in the third column. Interaction p-values are separately displayed for the elderly, the younger group, and the total sample. We discovered that even when data were stratified by the four seasons, the three months within each season still suggested mostly significant interactions between temperature measures and months (the seasonal effect) additionally adjusted for O_3_ exposure. Results additionally adjusted for PM_10_ instead of O_3_ were similar and thus the results are not shown. As a consequence, the adjustment for the seasonal effect may mislead the associated findings. Thus, the monthly stratified analysis could better reduce the interaction between the seasonal effect and temperature, avoid erroneous estimates, and capture the fine patterns of association between temperature measures and mortality throughout the year. Therefore, all analyses were conducted in the monthly stratified manner as to better avoid the issue of heterogeneity caused by the seasonal effect.

**Table 1:** Month by temperature interaction by 4 seasons in the total, younger and elder population adjusted for O_3_ a.

		Interaction *P*-values											
Temperature measures	Season	Total Population				Younger Population				Elder Population			
		Lag 0	Lag 1	Lag 2	Lag 3	Lag 0	Lag 1	Lag 2	Lag 3	Lag 0	Lag 1	Lag 2	Lag 3
Mean	Spring	0.546	0.254	0.847	0.499	0.790	0.995	0.846	0.859	0.367	0.174	0.655	0.649
Mean	Summer	0.256	0.366	0.195	**0.001** *	**0.043** *	0.173	0.289	**0.001** *	0.154	0.056	0.382	0.151
Mean	Fall	0.060	**0.002** *	**0.011** *	**0.018** *	**0.018** *	**0.004** *	0.119	0.161	0.753	0.140	0.073	0.133
Mean	Winter	0.870	**0.021** *	**0.004** *	**0.001** *	**0.033** *	0.223	0.140	**0.041** *	0.345	0.076	**0.015** *	**0.011** *
Max	Spring	0.113	**0.025** *	0.561	0.105	0.856	0.661	0.696	0.609	**0.037** *	0.055	0.492	0.266
Max	Summer	0.431	0.952	0.220	**0.002** *	0.080	0.085	0.369	**0.003** *	0.474	0.189	0.516	0.141
Max	Fall	**0.025** *	**0.041** *	0.118	**0.014** *	**0.005** *	**0.018** *	0.301	0.195	0.319	0.670	0.450	0.101
Max	Winter	0.277	0.152	**0.010** *	**0.001** *	**0.023** *	0.806	0.196	**0.045** *	0.917	0.166	0.053	**0.018** *
Min	Spring	0.979	0.549	0.469	0.526	0.723	0.898	0.982	0.947	0.944	0.331	0.479	0.599
Min	Summer	**0.020** *	0.108	0.080	**0.006** *	0.347	0.174	0.140	**0.003** *	**0.007** *	**0.039** *	0.273	0.376
Min	Fall	0.061	**0.027** *	**0.041** *	0.312	0.129	0.219	0.104	0.502	0.368	0.118	0.298	0.552
Min	Winter	0.250	**0.009** *	**0.035** *	**0.003** *	0.401	**0.030** *	0.189	0.055	**0.007** *	0.078	0.058	**0.033** *
Range	Spring	**0.010** *	**0.000** *	0.119	**0.000** *	0.907	0.083	0.404	0.372	**0.001** *	**0.004** *	0.077	**0.000** *
Range	Summer	0.169	0.102	0.837	0.104	0.172	**0.003** *	0.947	0.356	0.153	0.941	0.570	0.197
Range	Fall	0.206	0.140	0.374	0.071	0.084	0.141	0.804	0.366	0.367	0.236	0.485	0.134
Range	Winter	**0.004** *	0.472	0.492	0.386	0.054	0.302	0.538	0.639	**0.032** *	0.210	0.662	0.507

a Models were adjusted for daily mean of ozone exposure, city effects, calendar year, daily relative humidity, and holidays.

*P-value <0.05

All statistical analyses were performed using SAS 9.3 and p-values less than 0.05 were considered statistically significant.

## Results

The total population and total death count of Taiwan were 23,037,031 and 143,172 in 2008, according to the national statistics of the Taiwan Directorate General of Budget, Accounting and Statistics of the Executive Yuan. The study areas were 6 major cities of which the total population occupies 48.5% of the total population of Taiwan (Keelung 1.7%, Taipei 11.4%, Taichung 11.4%, Chiayi 1.2%, Tainan 8.1%, and Kaohsiung 12.0%). Furthermore, the total death count of six cities was 44.9% of the total for Taiwan (Keelung 1.8%, Taipei 10.9%, Taichung 9.7%, Chiayi 1.2%, Tainan 9.1%, and Kaohsiung 12.2%).

**Figure 2:**
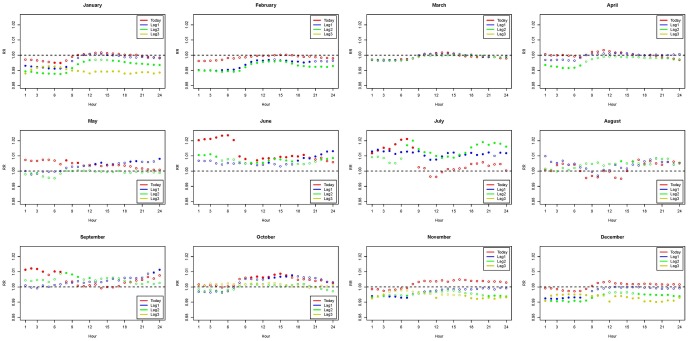
Distribution of hourly relative risk in the elderly population adjusted for O_3_. (Abbreviations: max, maximum; min, minimum; RR, relative risk; diff, daily range; T_5am_, temperature at 5 am; T_8am_, temperature at 8 am). Models were adjusted for daily mean of ozone exposure, city effects, calendar year, daily relative humidity, and holidays.

**Figure 3:**
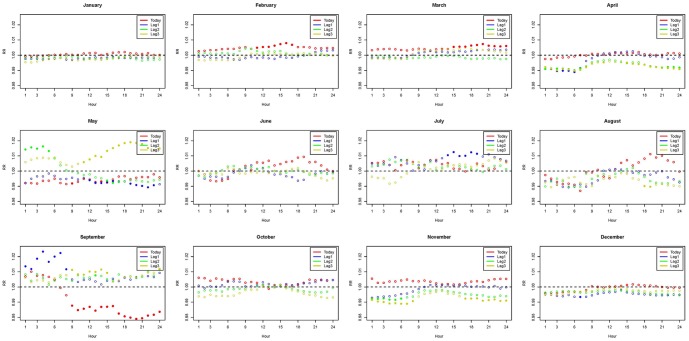
Distribution of hourly relative risk in the younger population adjusted for O_3_. (Abbreviations: max, maximum; min, minimum; RR, relative risk; diff, daily range; T_5am_, temperature at 5 am; T_8am_, temperature at 8 am.) Models were adjusted for daily mean of ozone exposure, city effects, calendar year, daily relative humidity, and holidays.

**Figure 4:**
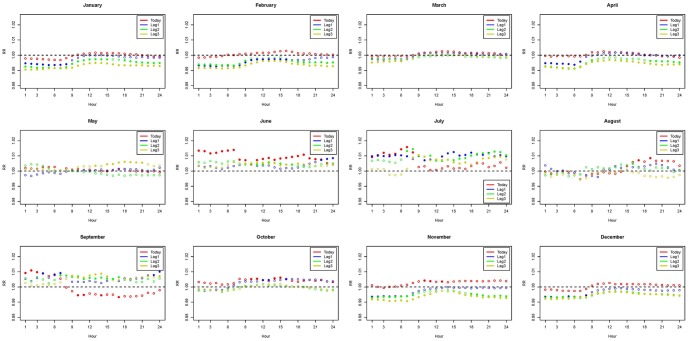
Distribution of hourly relative risk in the total population. (Abbreviations: max, maximum; min, minimum; RR, relative risk; diff, daily range; T_5am_, temperature at 5 am; T_8am_, temperature at 8 am.) Models were adjusted for daily mean of ozone exposure, city effects, calendar year, daily relative humidity, and holidays.

In Table 2, we present the associations between mortality and different temperature measures after controlling for potential confounding variables. “Mean”, “Max”, “Min”, and “Diff” represent the most significant daily statistics and the corresponding months. In an effort to provide an easier application and clearer presentation of the hourly temperature analysis, we selected “5 am” and “8 pm” instead of the most significant hours that vary slightly from month to month. The most significant hourly associations are very close to these two specific times. Regarding the issue of multiple testing due to 24 separate hourly models, we could see that most p-values in Table 2 survive the stringent Bonferroni's correction for multiple testing, which is the significance level being divided by the 24 hourly tests (0.05/24). In Figures 2 to 4, the relative highs or lows are mostly around 5 am and 8 pm. Results adjusted for PM_10_ are shown in the Table S2. The hourly associations are consistently good surrogates for daily analysis across the elderly, the younger group, and the total population. This phenomenon remains for the analysis using lag 0 to lag 3 temperature measures. We performed further analyses with more lagged hours and the results were similar to those shown in Table 2. Therefore, results of more lagged hourly measures are not included.

**Table 2:** Relative risk of temperature on mortality adjusted for O_3_ a.

	Elder			Younger			Total		
Temp.	month	RR	*P*-value	month	RR	*P*-value	month	RR	*P*-value
**Lag 0**									
**mean**	6	1.019	0.0008	9	0.985	0.0135	6	1.015	0.0007
**max**	10	1.007	0.0179	9	0.986	0.0014	6	1.007	0.0121
**min**	6	1.017	0.0006	2	1.005	0.1564	6	1.011	0.0051
**diff**	4	1.011	0.0002	9	0.980	0.0002	9	0.987	0.0001
**T_5am_**	6	1.023	<.0001	5	0.994	0.2235	9	1.007	0.057
**T_8am_**	7	1.015	0.0126	7	1.006	0.4555	7	1.012	0.0121
**Lag 1**									
**mean**	7	1.022	0.0003	5	0.990	0.0395	7	1.020	<0.0001
**max**	7	1.011	0.0111	5	0.990	0.0028	7	1.011	0.0012
**min**	2	0.991	0.0002	9	1.016	0.0086	2	0.994	0.0028
**diff**	10	1.013	0.0004	4	1.012	0.001	4	1.007	0.0017
**T_5am_**	2	0.990	<.0001	4	0.990	0.005	2	0.993	0.0005
**T_8am_**	2	0.992	0.0003	9	1.012	0.034	2	0.993	0.0006
**Lag 2**									
**mean**	7	1.023	<.0001	4	0.992	0.021	7	1.017	<0.0001
**max**	7	1.014	0.0003	9	1.008	0.0526	7	1.010	0.0008
**min**	1	0.988	<.0001	4	0.989	0.003	2	0.991	<0.0001
**diff**	6	1.009	0.035	2	1.008	0.0162	9	1.008	0.012
**T_5am_**	1	0.988	<.0001	5	1.013	0.0098	1	0.992	<0.0001
**T_8am_**	1	0.988	<.0001	4	0.993	0.0148	1	0.992	<0.0001
**Lag 3**									
**mean**	2	0.989	<.0001	5	1.015	0.0009	1	0.991	<0.0001
**max**	2	0.994	<.0001	5	1.009	0.007	1	0.994	<0.0001
**min**	1	0.988	<.0001	4	0.989	0.0032	2	0.991	<0.0001
**diff**	7	1.013	0.0015	3	1.008	0.017	7	1.011	0.0006
**T_5am_**	1	0.988	<.0001	11	0.989	0.0043	1	0.991	<0.0001
**T_8am_**	1	0.988	<.0001	4	0.992	0.0086	1	0.991	<0.0001

Abbreviation: max, maximum; min, minimum; RR, relative risk; diff, daily range; T_5am_, temperature at 5 am; T_8am_, temperature at 8 am.

a Models were adjusted for daily mean of ozone exposure, city effects, calendar year, daily relative humidity, and holidays.

The patterns of associations in 24 hours adjusted for daily mean ozone exposure are presented in Figure 2, Figure 3, and Figure 4 for the elderly, the younger group, and the total population, respectively. Figures adjusted for the daily maximum of PM_10_ are provided in the Figures S1–S3. Relative risks that are statistically significant are in dots, while non-significant relative risks are in circles. Relative risk of 1 is the reference line indicating no association between the hourly measures and daily mortality. The associations are mostly significant in the colder months (November - February) and some are significant in the hotter months (June - September). In all figures, increasing hourly mean temperature, from lag 0 to lag 3 measures, consistently reveals a protective effect for human health in colder months since the relative risks are below that reference line. However, higher hourly mean temperatures show an adverse effect on human health in hotter months, because the relative risks generally exceed 1. Comparing Figure 2 to Figure 3, we see that the elders are more affected by temperature variations comparing to the younger population. Figure 4 shows the mixed information from Figure 2 and Figure 3. In the significant associations, the early morning around 5 am and the night time around 8 pm show the greatest impact on mortality. Therefore, the results suggest that elders in particular should be more aware of temperature variations in the early morning and night time.

## Discussion

To date, hourly temperature has not been modeled for daily mortality. It is worth noting that hourly temperature models conveyed somewhat different messages other than the daily analysis. Daily temperature models attempt to describe the association between daily mortality and temperature [21]-[24], while hourly temperature models not only assess such associations, but also indicate the specific time of day that affects human health the most.

Our study finds that temperature in the early morning (e.g. 5AM), which is usually the lowest daily temperature, is significantly associated with daily mortality. This finding is consistent with previous studies that have found that extreme temperatures are associated with mortality [25]–[29]. One previous study also found that falls in ambient temperature contribute to excessive cardiovascular-related mortality [29]. This could further verify the strong association between early morning temperature with daily mortality, since people may experience dramatic decreases in ambient temperature when exposed to relatively cold outdoor temperatures compared with indoor ones when engaged in certain activities, such as commuting by motorcycle or outdoor morning exercise. In addition, the night time around 8 pm also revealed significant associations. Our hypothesis is that when people are getting out of bed or going to bed, they wear the minimum clothes and hence embrace the most impact from temperature variations, especially the elderly whose sleep pattern is associated with health [30–32].

This study adopts monthly stratified analysis and investigates the various impacts of both daily and hourly temperature measures on daily mortality in different populations. In the elderly population, without the considerations of hourly temperature, the daily range of temperature had the most significant impact on mortality in both spring and fall, while the mean, minimum and maximum temperature measures were strongly associated with mortality in winter and summer. The analysis of hourly temperature data provides the most significant associations (the smallest p-values) in most scenarios. This phenomenon is similar in the younger population, who are affected the most by temperature measures in hot months. Analyses show mixed information for the total population, since the older populations were affected by temperature differently comparing to the younger population. Nevertheless, the hourly temperature measures outperformed the daily statistics in most situations and the most crucial time of day that affects human health are quite consistent. A previous study reported the U-shape relationship between temperature and all-cause mortality among the Taiwanese elderly population, where the natural cubic spline, which is a widely used statistical technique in time-series analyses, was implemented to control for the unmeasured/residual confounding effects using calendar time as a proxy [21]. However, the natural cubic spline assumes a smoothing relationship between calendar time and mortality. Thus, this study may be limited by imperfect controls for monthly confounding as well as ignorance of monthly modifications in temperature-mortality relationship. There are also more reports suggesting a non-linear association between temperature and mortality [26], [27]. In contrast, our study assesses the association between temperature and mortality by monthly stratification so as to minimize the confounding effects of calendar time and to allow for the heterogeneous association between temperature and all-cause mortality. We assume a linear-relationship between the daily/hourly temperature measures and mortality given the small range of temperature measures within each month, since the daily temperature measures and the high resolution of hourly temperature measures demonstrate a somewhat linear relationship with daily mortality.

Some of the limitations of this study are mentioned as follows. First, some important factors that vary significantly by city may not be fully adjusted in the regression models that incorporate the city as a covariate. Second, the misclassification of temperature measurements may overestimate or underestimate the association with short-term mortality based on the scenario of exposure misclassification (differential or non-differential settings).

The strengths of this study include: 1) monthly stratified analysis is influenced less by the effect modification of seasons on the association between temperature measures and daily mortality when compared to other strategies such as seasonal adjustments or stratifications, 2) fine resolution of hourly temperature measures provides similar or even stronger association with daily mortality, 3) the proposed method discovered the most crucial time of a day that affects human health and various patterns of associations from January through December.

Based on the results, it is recommended that the public could be more aware of the temperature forecast for the early morning around 5 am and night time around 8 pm, rather than just focusing on the daily mean, minimum, maximum and range.

## Supporting Information

Figure S1 **Relative risk of hourly temperature in the elderly population adjusted for PM_10_.** Models were adjusted for daily maximum PM_10_, city effects, calendar year, daily relative humidity, and holidays.
(DOCX)

Figure S2 **Relative risk of hourly week in the younger population adjusted for PM_10_.** Models were adjusted for daily maximum PM_10_, city effects, calendar year, daily relative humidity, and holidays.
(DOCX)

Figure S3 **Relative risk of hourly temperature in the total population adjusted for PM_10_.** Models were adjusted for daily maximum PM_10_, city effects, calendar year, daily relative humidity, and holidays.
(DOCX)

Table S1 **Sensitivity analysis of O_3_ using the daily mean temperature in the elderly population.**
(DOCX)

Table S2 **Relative risk of temperature on mortality adjusted for PM_10_.**
(DOCX)
