# Supplementary material for: When Are We Most Vulnerable to Temperature Variations in a Day?
Source: PLoS One. 2014 Dec 2;9(12):e113195. doi: 10.1371/journal.pone.0113195 (PMC4251982; doi:10.1371/journal.pone.0113195)
Supplement: Figure S3 — Relative risk of hourly temperature in the total population adjusted for PM10. Models were adjusted for daily maximum PM10, city effects, calendar year, daily relative humidity, and holidays. (DOCX) [file pone.0113195.s003.docx]

**Figure S3. Relative risk of hourly temperature in the total population adjusted for PM_10_.** Models were adjusted for daily maximum PM_10_, city effects, calendar year, daily relative humidity, and holidays.

**
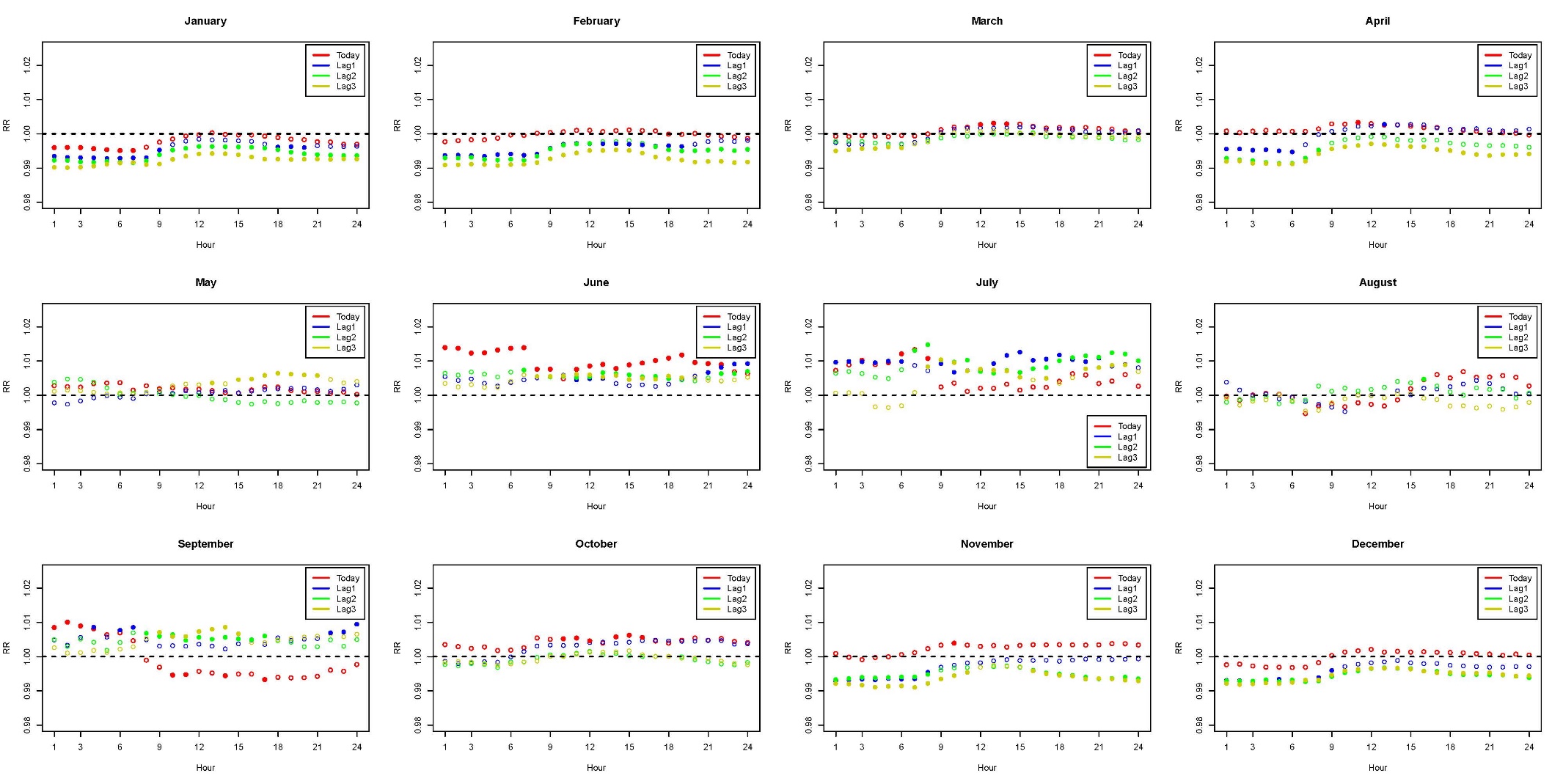
**
