# Supplementary material for: When Are We Most Vulnerable to Temperature Variations in a Day?
Source: PLoS One. 2014 Dec 2;9(12):e113195. doi: 10.1371/journal.pone.0113195 (PMC4251982; doi:10.1371/journal.pone.0113195)
Supplement: Table S1 — Sensitivity analysis of O3 using the daily mean temperature in the elderly population. (DOCX) [file pone.0113195.s004.docx]

**Table S1. Sensitivity analysis of O_3_ using the daily mean temperature in the elderly population**

| **Obs** | **Month** | **Parameter** | **Level1** | **DF** | **Estimate** | **StdErr** | **LowerWaldCL** | **UpperWaldCL** | **ChiSq** | **ProbChiSq** |
| --- | --- | --- | --- | --- | --- | --- | --- | --- | --- | --- |
| ***Max O_3_*** | | | | | | | | | | |
| **5** | 5 | Daily_T_Mean |  | 1 | 0.0077 | 0.0036 | 0.0008 | 0.0147 | 4.71 | 0.0300 |
| **6** | 6 | Daily_T_Mean |  | 1 | 0.0193 | 0.0053 | 0.0089 | 0.0297 | 13.18 | 0.0003 |
| **7** | 7 | Daily_T_Mean |  | 1 | 0.0145 | 0.0089 | -0.0030 | 0.0319 | 2.64 | 0.1042 |
| ***Mean O_3_*** | | | | | | | | | | |
| **5** | 5 | Daily_T_Mean |  | 1 | 0.0059 | 0.0038 | -0.0016 | 0.0134 | 2.37 | 0.1235 |
| **6** | 6 | Daily_T_Mean |  | 1 | 0.0185 | 0.0055 | 0.0077 | 0.0292 | 11.28 | 0.0008 |
| **7** | 7 | Daily_T_Mean |  | 1 | 0.0155 | 0.0090 | -0.0022 | 0.0332 | 2.94 | 0.0862 |

Models were further adjusted for city effects, calendar year, daily relative humidity, and holidays.
