# Supplementary material for: When Are We Most Vulnerable to Temperature Variations in a Day?
Source: PLoS One. 2014 Dec 2;9(12):e113195. doi: 10.1371/journal.pone.0113195 (PMC4251982; doi:10.1371/journal.pone.0113195)
Supplement: Table S2 — Relative risk of temperature on mortality adjusted for PM10. (DOCX) [file pone.0113195.s005.docx]

**Table S2. Relative risk of temperature on mortality adjusted for PM_10_^a^**

|  | **Elder** | | | **Younger** | | | **Total** | | |
| --- | --- | --- | --- | --- | --- | --- | --- | --- | --- |
| **Temp** | **month** | **RR** | **P-value** | **month** | **RR** | **P-value** | **month** | **RR** | **P-value** |
| **Lag 0** | | | | | | | | | |
| **mean** | 6 | 1.020 | 0.0002 | 9 | 0.985 | 0.0151 | 6 | 1.016 | 0.0003 |
| **max** | 6 | 1.008 | 0.0207 | 9 | 0.987 | 0.0027 | 6 | 1.008 | 0.0048 |
| **min** | 6 | 1.019 | 0.0002 | 3 | 1.004 | 0.1879 | 6 | 1.011 | 0.0042 |
| **diff** | 4 | 1.012 | 0.0001 | 9 | 0.981 | 0.0007 | 9 | 0.987 | 0.0002 |
| **T_5am_** | 6 | 1.024 | <.0001 | 9 | 1.005 | 0.3981 | 6 | 1.013 | 0.0008 |
| **T_8am_** | 7 | 1.011 | 0.0075 | 5 | 0.991 | 0.0537 | 6 | 1.008 | 0.0235 |
| **Lag 1** | | | | | | | | | |
| **mean** | 7 | 1.021 | 0.0006 | 7 | 1.016 | 0.0492 | 7 | 1.019 | <.0001 |
| **max** | 6 | 1.007 | 0.0213 | 5 | 0.991 | 0.0051 | 7 | 1.010 | 0.0018 |
| **min** | 1 | 0.991 | 0.0002 | 9 | 1.014 | 0.0215 | 1 | 0.994 | 0.0014 |
| **diff** | 10 | 1.012 | 0.001 | 4 | 1.013 | 0.0006 | 4 | 1.008 | 0.0005 |
| **T_5am_** | 1 | 0.990 | <.0001 | 4 | 0.990 | 0.0052 | 1 | 0.993 | 0.0002 |
| **T_8am_** | 1 | 0.990 | 0.0001 | 12 | 0.993 | 0.0472 | 1 | 0.993 | 0.0005 |
| **Lag 2** | | | | | | | | | |
| **mean** | 7 | 1.023 | <.0001 | 4 | 0.993 | 0.0266 | 7 | 1.016 | 0.0001 |
| **max** | 7 | 1.013 | 0.0009 | 9 | 1.007 | 0.079 | 7 | 1.010 | 0.0015 |
| **min** | 1 | 0.988 | <.0001 | 4 | 0.989 | 0.0033 | 2 | 0.991 | <.0001 |
| **diff** | 6 | 1.008 | 0.02 | 9 | 1.009 | 0.0696 | 9 | 1.008 | 0.0135 |
| **T_5am_** | 1 | 0.988 | <.0001 | 5 | 1.013 | 0.0104 | 1 | 0.992 | <.0001 |
| **T_8am_** | 1 | 0.988 | <.0001 | 4 | 0.993 | 0.0172 | 1 | 0.992 | <.0001 |
| **Lag 3** | | | | | | | | | |
| **mean** | 2 | 0.988 | <.0001 | 5 | 1.015 | 0.0009 | 1 | 0.991 | <.0001 |
| **max** | 2 | 0.993 | <.0001 | 5 | 1.009 | 0.0068 | 1 | 0.994 | <.0001 |
| **min** | 1 | 0.989 | <.0001 | 4 | 0.989 | 0.0029 | 2 | 0.990 | <.0001 |
| **diff** | 7 | 1.013 | 0.0019 | 3 | 1.008 | 0.0102 | 7 | 1.011 | 0.0006 |
| **T_5am_** | 1 | 0.988 | <.0001 | 11 | 0.989 | 0.0052 | 1 | 0.991 | <.0001 |
| **T_8am_** | 1 | 0.988 | <.0001 | 4 | 0.992 | 0.0086 | 1 | 0.991 | <.0001 |

Abbreviation: max, maximum; min, minimum; RR, relative risk; diff, daily range; T_5am_, temperature at 5 am; T_8am_, temperature at 8 am.

^a^ Models were adjusted for daily maximum PM_10_, city effects, calendar year, daily relative humidity, and holidays.
